# Implementation of Integrated Learning Program in neurosciences during first year of traditional medical course: Perception of students and faculty

**DOI:** 10.1186/1472-6920-8-44

**Published:** 2008-09-24

**Authors:** Sarmishtha Ghosh, Himanshu V Pandya

**Affiliations:** 1 Department of Physiology, Pramukhswami Medical College, Karamsad, Gujarat, India; 2 Department of Medicine, Pramukhswami Medical College, Karamsad, Gujarat, India; 3 Current address: Department of Physiology, Faculty of Medicine, MAHSA College, Jalan University Campus, Kuala Lumpur, Malaysia

## Abstract

**Background:**

Our college introduced an integrated learning program (ILP) for first year undergraduates with an aim to develop, implement and evaluate a module for CNS in basic sciences and to assess the feasibility of an ILP in phase I of medical education in a college following traditional medical curriculum.

**Methods:**

The idea of implementing ILP for Central Nervous System (CNS) in phase one was conceived by curriculum development committee drawn from faculty of all phases. After a series of meetings of curriculum development committee, inputs from basic science and clinical departments, a time table was constructed. Various teaching learning methods, themes for integrated didactic lectures, case based learning and clinical exposure were decided. Basic science faculty were made to participate actively in both case based learning and hospital visits along with clinical experts. The completed program was evaluated based on structured questionnaire.

**Results:**

Sixty percent students rated the program good to excellent with reference to appreciation, understanding and application of basic science knowledge in health and disease. Seventy eight percent felt that this program will help them perform better in later days of clinical training. However sixty percent students felt that ILP will not help them perform better at the first professional examination. Seventy two per cent of faculty agreed that this program improved understanding and application of basic science knowledge of students. Ninety percent of faculty felt that this program will help them perform better in later days of clinical training.

**Conclusion:**

The adoption of present integrated module for CNS and the use of multiple teaching learning methods have been proven to be useful in acquisition of knowledge from the student satisfaction point of view. Students and faculty expressed an overall satisfaction towards ILP for CNS. The study showed that it is possible to adopt an integrated learning module in the first year of medical course under a conventional curriculum.

## Background

Changing needs of the society, advances in scientific knowledge, and innovations in the educational field necessitate constant changes in medical school curricula. Various innovations and trends which have been undertaken globally include education for capability, community orientation in medical education, self directed learning, problem based learning, integration and early patient contact. An integrated medical curriculum refers to a non compartmentalized approach to basic sciences whereby lectures on subjects like embryology, histology, anatomy, physiology and pathology, are alternated over the course of first two years [1], organized around organ systems such as cardiovascular or nervous with a major component of problem based learning. GPEP report [2], ACMI-TRI project report [3] and recommendations of General Medical Council, UK [4] propose the need for greater integration of subjects in the medical curriculum.

Medical colleges in India have been following a traditional curriculum, characterized by "discipline wise model" with a high degree of compartmentalization into subjects of basic sciences, paraclinical and clinical branches. Several areas of redundancy, repetition and overlapping along with the observation of a gap between the qualitative and quantitative advancement in medical education and achievements in the field of health care prompted the Medical Council of India to adopt a need based curriculum for undergraduate medical education in India. "Regulations on Graduate Medical Education, 1997" recommend a teaching approach characterized by maximal efforts to encourage integrated teaching between traditional subject areas using a problem based learning approach and de-emphasize compartmentalization of disciplines so as to achieve both horizontal and vertical integration in different phases [5].

Under the existing system of undergraduate curriculum at our college, Central Nervous System (CNS) was taught for many years in a non integrated, discipline based manner wherein the three preclinical departments of Anatomy, Physiology and Biochemistry taught their respective subjects primarily through didactic lectures interspersed with tutorials on case based learning format, laboratory practical exercises and group seminars on related clinical topics. The number of hours were stipulated to respective departments and there was no horizontal or vertical integration other than inputs by clinician after the seminars presented by the students on some occasions.

Our college, recognized by the Medical Council of India, in pursuit of current recommendations of MCI planned to introduce an Integrated Learning Program (ILP) for the preclinical phase (Phase I) of undergraduate medical education and subsequently in paraclinical (Phase II) and clinical (Phase III) phases. In phase I, the topic of CNS was chosen.

The objective was to develop and implement a module for CNS in basic sciences which would incorporate and focus on integrated learning using multiple teaching methodologies. It also aimed to assess the feasibility and importance of an ILP in phase I of medical education in a college following traditional medical curriculum.

## Methods

### Framing of time table

A curriculum committee was formed with a core group of faculty from basic, paraclinical and clinical departments. Series of meetings were conducted to discuss the feasibility of introducing an ILP during phase I of Bachelor of Medicine, Bachelor of Surgery [MBBS] course. An external faculty from Christian Medical College, Vellore with experience of implementing ILP in basic sciences shared her experience with the faculty of basic science departments during her visit to conduct a workshop on problem based learning. Thereafter, it was decided to implement a module of ILP of 6-weeks duration for the topic of CNS involving the departments of Anatomy, Physiology and Biochemistry. Since teaching -learning of CNS involves significant integration of structure and function, it was chosen as the topic of ILP module. Incidentally this was also the only system remaining to be covered for the batch of 2006–07 before their first professional examinations.

The entire faculty of departments of Anatomy, Physiology and Biochemistry were oriented to the process of implementing an ILP and the learning objectives were decided after discussions amongst the basic science faculty and curriculum committee members. Attempts were made to ensure time integration of the different topics horizontally as well as vertically.

Various teaching learning methods were decided to ensure active participation from the students and also improve their analytical and clinical reasoning skills. This was done with the objective of making them understand and apply the basic science concepts in health and disease better and in a setting of clinical relevance. The teaching learning methods incorporated were [a] didactic lectures, [b] case stimulated interactive lectures, [c] case based group learning, [d] student group seminars, [e] dissection, [f] practicals/demonstrations and [g] patient exposure.

**Figure 1:**
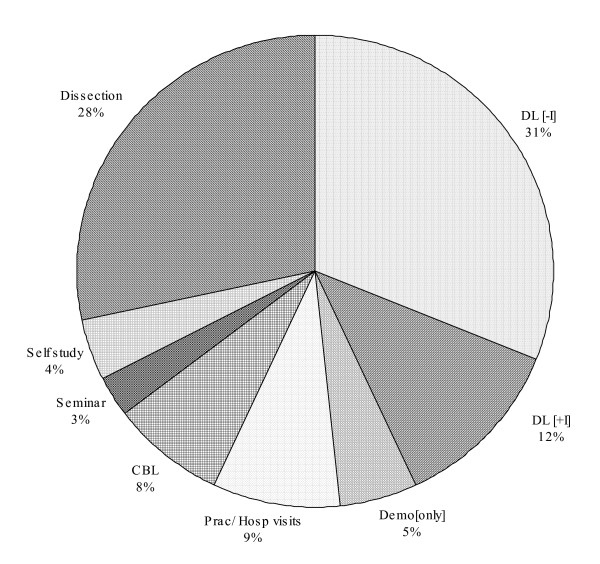
**Distribution of various teaching learning methods employed in ILP module.** Total number of hours = 199; DL [-I]- Didactic Lecture without interaction, DL [+I]- Interactive lectures (Case stimulated), CBL – Case based Learning, Demo [only]-demonstration without hands on exercise.

The proportion of each method is given in Figure 1.

### Implementation of time table

The program started with an orientation class with the objective of making the students aware of the process and relevance of ILP in undergraduate medical course.

The topics were delivered by means of the various teaching learning methods as stated earlier and are described below.

A] Didactic Lectures: This approach consisted of large group lectures delivered by faculty of basic science departments in a traditional fashion on different components of CNS, intending to give an idea of the basic concepts to the students.

B] Case stimulated Interactive Lectures: This approach employed large group lecture either in a quiz fashion or with a patient problem accompanied by questions. Such interactive stimulatory method ensured highlighting of the basic science concepts by the teacher based on student responses. Some lectures included presentation of a case scenario followed by group discussions amongst students and final summing up by the faculty, clearly stating the learning objectives expected to be known to students. This ensured active learning by the students and promoted their reasoning ability in the setting of large group lecture.

C] Case Based Learning: Written case scenarios based on real patients were presented to the students. Each case was accompanied by questions to stimulate a focused enquiry and self directed learning. Small group discussions of 1 hour were supervised by basic science faculty followed by a wrap up session in large group conducted by a clinician teacher.

D] Group Seminars: Topics of CNS of clinical relevance were assigned to a batch of 5 students who prepared a seminar under supervision of a basic science faculty and presented the topic in the large class. The entire session was of 1 hour duration where each student presented and interacted with the large group. This was followed by a summing up session by a faculty from the relevant clinical discipline. The seminars were intended to induce active participation from the students and also to improve communication and presentation skills.

E] Patient Exposure: Students accompanied by a basic science faculty visited hospital wards for early clinical exposure. The hospital visits were conducted on three days of a week and all 100 students got a chance to have patient contact over the whole week. Each batch of 33 students was further divided into two batches of 15–16 students per group. They were exposed to clinical cases which were prototype neurological disorders and most of them were actual cases on whom case based learning occurred earlier. This was planned to ensure that students have better understanding of the clinical features and allied discussions on the patients after having undergone the case based learning sessions. The bed side discussion included patient history, symptoms, physiological basis of patient condition and the treatment in brief. The discussion also incorporated other non scholastic features of medical profession such as doctor patient interaction and communication skills.

### Student feedback

After completion of the module, feedback was collected on the last day, using questionnaires. The questions were framed after having focus group discussions, keeping the following themes in mind: utility of ILP as an important means of teaching medical students with respect to understanding, appreciation and application of basic science knowledge to health and disease. It also aimed at rating of various teaching learning methods with respect to their importance in improving scholastic and non-scholastic facets of medical education. A 5-pt Likert scale with a score of 1 = poor, 2 = satisfactory, 3 = good, 4 = very good and 5 = excellent was used to find out the rating and a 3 point scale (1 = not at all, 2 = to some extent and 3 = to great extent) was used to elicit various responses from the students. The anonymous questionnaire also had a free comment section for narrative by the students. The free comments were analyzed by one author and following themes were identified: time table, CBL, Time of implementation, Interactive sessions and hospital visits, etc.

### Faculty Feedback

Questionnaire for faculty was designed with an aim to find out the level of satisfaction of faculty with activities related to planning and implementation of integrated program in CNS and their likes and dislikes for the program with regard to their change in attitude. The questionnaire had a free section for narrative comments which were thereafter analyzed. The questionnaire for the faculty was sent to individuals in closed envelope from the office of Dean with a request to return it confidentially.

[see Additional file 1]

The study was approved for conduct and publication by the 'Human Research Ethics Committee of Pramukhswami Medical College at H. M. Patel Center for Medical Care & Education' and the questionnaire presented to participants were preceded by explanation of its purpose and assurance of confidentiality of results. Detailed explanation of questionnaire was presented to the students by one of the authors. Since it was an anonymous questionnaire, written informed consent was not obtained and response to questionnaire was implied as verbal consent. A different anonymous questionnaire was sent to the faculty members of basic science departments through the dean's office and their response was implied as consent.

## Results

**Figure 2:**
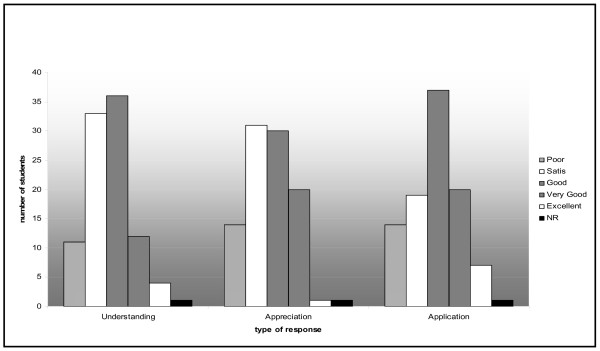
Overall rating of Integrated Learning Program on Central Nervous System with regard to understanding, appreciation and application of basic knowledge of nervous system to health and disease; N = 98.

Table 1 shows that 78% students felt that the ILP would be beneficial for them to perform better in the later days of clinical exposure while 36% felt that it would help in University Examination of preclinical phase. 86% faculty agreed that ILP will definitely improve the performance of students in later days of clinical training 43% were positive about performance in University professional examination. Many faculty were uncertain about these responses. Figure 2 shows that there has been an overall satisfaction amongst the first year students with regard to ILP in CNS as a useful module for understanding, appreciation and application of basic science knowledge to health and disease. The variety of teaching learning methods have been appreciated in a differential manner by the students and the active learning strategies, namely, case stimulated interactive lectures, group seminars and hospital visits for early clinical exposure were rated better by the students while the didactic lectures and case based learning were less appreciated by them. [Table 2]. Most faculty expressed satisfaction to great extent with activities during the planning, framing and implementation of ILP namely framing of time table and delivery of content. Less number of faculty were involved in activities like assessment of students and evaluation of the program and hence the level of satisfaction was only to some extent [Table 3]. Table 4 shows the responses of faculty regarding the various aspects of the process of implementation of the integrated module. 71.4% liked interdepartmental discussions, interaction between the basic and clinical disciplines along with the coordination and group activity during the process of planning and implementation of ILP. This resulted in 36% faculty strongly recommending ILP for all systems and 57% recommending it to some extent in phase I of medical curriculum. However, only 43% faculty liked the assessment process since it was an integrated type and most faculty were not aware of the type. The free comments made by students and faculty are listed in Table 5. It was observed that students were particularly satisfied with the program though they were not appreciative of the structuring of the time table and the slots provided for self learning sessions. The standards of the cases were stated to be higher for a first year undergraduate. The students also considered the assessment to be tough and stressful since they had to prepare for three subjects at a time. The faculty were concerned about the feasibility of this program in timely completion of the preclinical basic science course which is of only one year in the Indian system of medical education.

**Table 1:** Perception of students and faculty regarding utility of Integrated Learning Program with reference to future performance of the students.

Utility of ILP	Faculty Response			Student Response		
	Yes	No	Uncertain	Yes	No	Uncertain

Better performance in clinics	12 [86%]	0	2 [14%]	76 [78%]	16 [16%]	6 [6%]
Better performance in University Exam	6 [43%]	3 [21%]	5 [36%]	35 [36%]	59 [60%]	4 [4%]

Number of students = 98 [98% of potential respondents]; Number of faculty = 14 [87.5% of potential respondents]

**Table 2:** Ratings of different methods of teaching-learning incorporated in ILP [CNS] by the students.

	Method	Poor	Good	Excellent	No response
1	Didactic Lecture without interaction	20 [20.4%]	**68 [69.4%]**	4 [4.08%]	5 [5.1%]
2	Case stimulated Interactive Lectures	14 [14.3%]	**78 [79.6%]**	4 [4.08%]	2 [2.04%]
3	Case based Learning	**40 [40.8%]**	**56 [57.1%]**	2 [2.04%]	0
4	Student Group seminars	20 [20.4%]	**61 [62.2%]**	**15 [15.3%]**	2 [2.04%]
5	Patient exposure-Hospital visit	8 [8.16%]	**58 [59.2%]**	**28 [28.6%]**	2 [2.04%]
6	Practical Exercises in the laboratory	1 [1.02%]	**63 [64.3%]**	**20 **[20.4%]	11 [11.2%]
7	Demonstrations	19 [19.4%]	**66 [67.3%]**	5 [5.1%]	7 [7.14%]

[N = 98/100; 98%]

**Table 3:** Response of faculty regarding level of satisfaction with activities during planning and implementation of ILP.

	Activity	Great extent	Some extent	Not at all	No Resp
1	Framing of Timetable	6 [42.8%]	4 [28.6%]	4 [28.6%]	0
2	Delivery of Content	8 [57.1%]	5 [35.7%]	0	1 [7.14%]
3	Assessment of Students	2 [14.3%]	9 [64.3%]	1 [7.14%	2 [14.3%]
4	Evaluation of Program	0	1 [7.14%]	0	13 [92.8%]

N = 14 [87.5% of potential respondents]

**Table 4:** Response of faculty on likes and dislikes regarding the activities involved in the process of planning and implementation of ILP; N = 14

	Activity	Liked	Did not like	Uncertain	No Resp
1	Interdepartmental discussions amongst basic science faculty	10 [71.4%]	1 [7.14%]	3 [21.4%]	0
2	Interdepartmental discussions amongst basic and clinical science faculty	10 [71.4%]	1 [7.14%]	3 [21.4%]	0
3	Integrated assessment of students	6 [42.8%]	4 [28.6%]	2 [14.3%]	1 [7.14%]
4	Coordination and group activity during planning and implementation of ILP	10 [71.4%]	1 [7.14%]	3 [21.4%]	0

**Table 5:** Free comments given by the faculty and students regarding various aspects of ILP-CNS.

Themes	Student [60/98 = 61.3%]	Faculty [5/15 = 33%]
Usefulness of Integrated Learning Program	1. ILP is good. All systems should be integrated	1. Time integration is more important than compulsory integration of everything-some topics may be left alone
	2. It helped in gaining in much more knowledge.	2. Integrated sessions shall be useful provided the systems/areas having scope for integration are identified,
	3. Integration is not good, it disturbs our own schedule	3. Adopting a flexible hybrid system comprising of both the traditional and ILP would be appreciated
	4. OK for few systems but not for all	4. ILP is useful

Time of Implementation	1. System was nice but probably adopted in a very stressful manner at the wrong time.	
	2. ILP should not be kept at the end of the session, should be started early	

Time table	1. Needs to be structured properly and followed also	
	2. Two successive lectures should not be of the same teacher	
	3. Fours hours of continuous didactic lectures become stressful	

Mode of content delivery	1. Case discussions were too many and they were mostly of higher standard which we were unable to grasp	1. Less time to be allotted for case discussion, specifically in the first six months as it's a phase of transition
	2. Case based learning were good but needed more organization	2. Cases to be framed after identifying the learning objectives of first year students
	3. Extra stress on cases compromised with understanding of normal physiology	3. Discussion amongst clinical and basic science teachers is a must before case is presented to the students and adequate time for learning to be given
	4. Dissections were too many which could have been reduced so that we get time to study	
	5. The hospital visits and patient contact was interesting	

Integrated Assessment at the end of the module	1. Stressful	
	2. Examination should be conducted on the pattern of the University examination	

## Discussion and conclusion

The present system of education follows a building block principle where each subject has its own frame, restricted to one part of the course. The disadvantages of such a system are unnecessary repetition, disjointed approach to teaching creating confusion in student's mind leading to failure of grasping the subject of medicine as a whole. Curriculum integration has therefore evolved as an important strategy in medical education [6]. Various integrated medical curricula have been adopted by many medical schools all over the globe to ensure holistic approach rather than a fragmented one in medical education to encourage meaningful learning [7].

In the integrated model, courses that used to be taught as disciplines, such as histology, anatomy, and physiology, has been taught as part of the integrated teaching in a block frame of six weeks. Thus, during the CNS block, students learned the anatomy, microanatomy, and physiology of the central nervous system that they would have previously learned in separate courses. They were also introduced to clinical sciences while undertaking case based learning and hospital visits. The weekly schedule of students divided into the following activities that were repeated each week: lectures, small group sessions through case based learning and around the real patients (patient-centered learning, or PCL), the doctoring cluster (clinical skills acquisition, community practice placement, professional development, and student-selected electives), biopractical exercises, and independent study. Less percentage of students appreciated the case based learning in class room since there were a lack of trained facilitators and the students liked the bedside case discussions in the hospitals more than the class room discussions. Also they opined that the cases used were of higher standard for a first year undergraduate. This opinion was helpful in considering designing cases for subsequent batches.

The daily lectures were intended to provide a conceptual organizing framework for students rather than as a means of delivering detailed factual information. In addition to acquiring knowledge of the basic sciences, students also acquired competency in the core abilities through their learning activities. The ILP was perceived to be useful by majority of students and most faculty with regard to performance of students in later days of clinical exposure. However there was mixed response from students with regards to performance at University. The students' response was more of a speculation since they were not exposed to an University examination, it was more of a prediction rather than actual response. Interestingly, a sizable number of faculty were uncertain on this issue. The uncertainty of the faculty can be explained by the fact that the faculty were not trained in the new medical education processes and the University examination pattern was not changed from a traditional manner. But both faculty and students appreciated the program to be a successful attempt in terms of understanding and appreciation of basic science knowledge in the context of health and disease through an integrated learning program incorporating diverse teaching learning methods. The program brought about for the first time a coordinated approach to teaching and learning amongst the basic science faculty as well as between the basic and the clinical science faculty in our institution.

In India, some medical colleges have introduced integrated teaching program with student centered case based learning to enhance clinical learning [8,9]. Modules have been introduced in training of students in medical schools following traditional curriculum in the first year and also in clinical clerkship. Such programs have been found to enhance student knowledge and integration of that knowledge together with improved attitude towards medical education.

The present Integrated Learning Program on Central Nervous System is an innovative attempt to introduce horizontal and vertical integration in Phase I of traditional medical curriculum.

The lessons learnt during the planning and implementation of ILP for CNS has been translated to all systems taught in the first and second year of medical curriculum in the institution and can serve as a model to be adopted by other medical colleges in India.

The study had a number of limitations which included lack of expert facilitators for conducting case based learning sessions, framing of timetable to satisfy the requirement of Medical Council of India with regard to number of hours allotted to the three different preclinical subjects and ignorance of few faculty members.

## Conclusion

The study showed that it is possible to adopt an integrated learning module in the first year of medical teaching under a conventional curriculum. The adoption of present integrated module for Central Nervous System and the use of multiple teaching learning methods have been proven to be useful in acquisition of knowledge from the student satisfaction point of view. The faculty though not having prior exposure to such a system also appraised the method as an useful one.

## Competing interests

The authors declare that they have no competing interests.

## Authors' contributions

SG has designed the study and framed the evaluation questionnaire for acquisition of data and analysed and interpreted the data. She has also been involved in drafting the manuscript.  HVP has been involved in drafting the manuscript or revising it critically for important intellectual content and have given final approval of the version to be published.

## Pre-publication history

The pre-publication history for this paper can be accessed here:



## Supplementary Material

Additional file 1 Evaluation of integrated learning program in CNS for preclinical phase-2007. This is the questionnaire used to evaluate the learning program by taking the responses of the students as well as faculty. Click here for file
