# Supplementary material for: Implementation of Integrated Learning Program in neurosciences during first year of traditional medical course: Perception of students and faculty
Source: BMC Med Educ. 2008 Sep 24;8:44. doi: 10.1186/1472-6920-8-44 (PMC2569025; doi:10.1186/1472-6920-8-44)
Supplement: Additional file 1 — Evaluation of integrated learning program in CNS for preclinical phase-2007. This is the questionnaire used to evaluate the learning program by taking the responses of the students as well as faculty. [file 1472-6920-8-44-S1.doc]

***PRAMUKHSWAMI MEDICAL COLLEGE, Karamsad***

**Evaluation of**

**INTEGRATED LEARNING PROGRAM IN CNS FOR PRECLINICAL PHASE - 2007**

*Dear Students,*

*this is an* ***ANNONYMOUS QUESTIONNAIRE*** *to evaluate your reaction towards implementation of an* ***Integrated Learning Program [ILP] Module in CNS***  *delivered by different teaching -learning methods namely Didactic Lecture [DL] without interaction, DL with interaction, Group Seminars, Demonstration with only observation, Demo. with observation and hands on experience ,Dissection, Case Based Learning ,Hospital Visit without Patient Contact and Hospital Visit with Patient Contact.*

***Your frank opinion will be highly appreciated. It will take not more than 15 minutes and the result will be kept confidential and will be pooled for data analysis in case it is proposed to be published.***

1. Rate the following teaching/learning methods used during the integrated learning program in CNS

1= Poor 2= Satisfactory 3= Good 4= Very Good 5= Excellent

A]Didactic Lecture

a) With interaction 1 2 3 4 5

b) Without interaction 1 2 3 4 5

B]Group seminars 1 2 3 4 5

C] Demonstration

a) Only observation 1 2 3 4 5

b) Observation + hands 1 2 3 4 5

on experience

D] Case Based Learning [CBL] 1 2 3 4 5

E] Hospital Visit

a) Without patient contact 1 2 3 4 5

b) With patient contact 1 2 3 4 5

2. Rate the overall ILP in CNS with regard to the following ( Scale same as above)

A] Understanding of 1 2 3 4 5

structure & function

relationship of CNS

B] Appreciation of basic 1 2 3 4 5

Sciences knowledge In

Health and disease

C].Application of knowledge 1 2 3 4 5

Of principles of basic Science

In health & disease

3.A) Rate the Teaching / Learning methods mentioned below with regard to understanding of structure / function relationship of CNS.

**1= Poor, 2=Satisfactory, 3=Good 4=V.Good 5= Excellent**

1 Didactic Lecture without interaction 1 2 3 4 5

2 DL. with interaction 1 2 3 4 5

3 Group Seminars 1 2 3 4 5

4. Demo. With only observation. 1 2 3 4 5

5. Demo. Obsv +hands on. experience 1 2 3 4 5

6. Dissection 1 2 3 4 5

7. CBL 1 2 3 4 5

8. Hospital Visit without Pt contact 1 2 3 4 5

9. Hospital Visit + Pt Contact. 1 2 3 4 5

B) Rate the T/L Methods mentioned below with regard to appreciation of basic science

knowledge in health disease.

**1= Poor, 2=Satisfactory, 3=Good 4=V.Good 5 = Excellent**

1 Did .L. without interaction 1 2 3 4 5

2 DL. with interaction 1 2 3 4 5

3 Group Seminars 1 2 3 4 5

4. Demo. with only observation 1 2 3 4 5

5. Demo. Obsr +hands on experience 1 2 3 4 5

6. Dissection 1 2 3 4 5

7. CBL 1 2 3 4 5

8. Hospital Visit without Pt contact 1 2 3 4 5

9. Hospital Visit + Pt Contact. 1 2 3 4 5

C) Rate the T/L Methods mentioned below with regard to application of Knowledge of principles of basic science in health & disease

**1= Poor, 2=Satisfactory 3=Good 4=V.Good 5= Excellent**

1 Did .L. without interaction 1 2 3 4 5

2 DL. with interaction 1 2 3 4 5

3 Group Seminars 1 2 3 4 5

4. Demo. With only observation 1 2 3 4 5

5. Demo. Obsr +hands on experience 1 2 3 4 5

6. Dissection 1 2 3 4 5

7. CBL 1 2 3 4 5

8. Hospital Visit without Pt contact 1 2 3 4 5

9. Hospital Visit + Pt Contact. 1 2 3 4 5

1. A) Which of the following T/L methods helped you to **become an active learner** ? Rate accordingly.

**1. Not at all 2. To some extent 3. To a great extent**

1. a) didactic lecture without interaction 1 2 3
2. b) didactic lecture with interaction 1 2 3
3. c) group seminar 1 2 3
4. d) Demo. only observation. 1 2 3
5. e) Demo. Obsr +hands on experience 1 2 3
6. f) Dissection 1 2 3
7. g) CBL 1 2 3
8. h) Hospital Visit without Pt contact 1 2 3
9. i)Hospital Visit + Pt Contact. 1 2 3
10. B) Which of the following T/L methods helped you to **become a motivated self directed learner.**
11. a) didactic lecture without interaction 1 2 3
12. b) didactic lecture with interaction 1 2 3
13. c) group seminar 1 2 3
14. d) Demo. only observation 1 2 3
15. e) Demo. Obsv +hands on experience 1 2 3
16. f) Dissection 1 2 3
17. g) CBL 1 2 3
18. h) Hospital Visit without Pt contact 1 2 3
19. i) Hospital Visit + Pt Contact. 1 2 3

C) Which of the following T/L methods enabled you to **learn the skill of working in group.**

1. a) didactic lecture without interaction 1 2 3
2. b) didactic lecture with interaction 1 2 3
3. c) group seminar 1 2 3
4. d) Demo. only obs. 1 2 3
5. e) Demo. Obsv +hands on experience 1 2 3
6. f) Dissection 1 2 3
7. g) CBL 1 2 3
8. h) Hospital Visit without Pt contact 1 2 3

i) Hospital Visit + Pt Contact. 1 2 3

.

. D) Which of the following T/L methods enabled you to **learn the skill of Presentation**

1. a) didactic lecture without interaction 1 2 3
2. b) didactic lecture with interaction 1 2 3
3. c) group seminar 1 2 3
4. d) Demo. only observation 1 2 3
5. e) Demo. Obsv+hands on experience 1 2 3
6. f) Dissection 1 2 3
7. g) CBL 1 2 3
8. h) Hospital Visit without Pt contact 1 2 3

i)Hospital Visit + Pt Contact. 1 2 3

E) Which of the following T/L methods enabled you to **learn skill of critical Reasoning**

1. a) didactic lecture without interaction 1 2 3
2. b) didactic lecture with interaction 1 2 3
3. c) group seminar 1 2 3
4. d) Demo. only observation 1 2 3
5. e) Demo. Obsr +hands on experience 1 2 3
6. f) Dissection 1 2 3
7. g) CBL 1 2 3
8. h) Hospital Visit without Pt contact 1 2 3

i) Hospital Visit + Pt Contact. 1 2 3

1. F) Which of the following T/L methods enabled you to **learn skill of interaction during presentation**
2. **[ e.g.facing questions or participating in discussion]**
3. a) didactic lecture without interaction 1 2 3
4. b) didactic lecture with interaction 1 2 3
5. c) group seminar 1 2 3
6. d) Demo. only observation 1 2 3
7. e) Demo. Obsv +hands on experience 1 2 3
8. f) Dissection 1 2 3
9. g) CBL 1 2 3
10. h) Hospital Visit without Pt contact 1 2 3
11. i) Hospital Visit + Pt Contact. 1 2 3
13. G) Which of the following T/L methods enabled you to **learn Skill of accessing & managing Information.**
14. a) didactic lecture without interaction 1 2 3
15. b) didactic lecture with interaction 1 2 3
16. c) group seminar 1 2 3
17. d) Demo. only observation 1 2 3
18. e) Demo. Obsv +hands on experience 1 2 3
19. f) Dissection 1 2 3
20. g) CBL 1 2 3
21. h) Hospital Visit without Pt contact 1 2 3

i) Hospital Visit + Pt Contact. 1 2 3

1. A] Do you think this ILP will help you to **perform better in University exam** Yes No

B] Do you think this ILP will help you to **perform better** **in later days of your clinical course**  Yes No

OPEN FORUM :

|  |
| --- |

***PRAMUKHSWAMI MEDICAL COLLEGE, Karamsad***

**Evaluation of**

**INTEGRATED LEARNING PROGRAM IN CNS FOR PRECLINICAL PHASE - 2007**

*Dear Faculty*

*This is an* ***ANNONYMOUS QUESTIONNAIRE*** *to evaluate your reaction towards implementation of an* ***Integrated Learning Program [ILP] Module in CNS***  *delivered by different teaching -learning methods namely Didactic Lecture [DL] with and without interaction, Group Seminars, Demonstration with only observation and with observation plus hands on experience ,Dissection, Case Based Learning ,Hospital Visit with and without Patient Contact.*

***Your frank opinion will be highly appreciated. It will take not more than 10 minutes and the result will be kept confidential and will be pooled for evaluation of Integrated Learning Program in CNS. You are also requested to please maintain confidentiality of your own response.***

***Please send back this feedback to Dean's Office latest by Monday, 27^th^ August, 2007 12-00 Noon***

***Thank you for your cooperation***

1. Please state the **degree of your involvement** for the activities during the planning and implementation of the Integrated Learning Program: [ tick the appropriate column]

|  | Item | To great extent | To some extent | Not at all |
| --- | --- | --- | --- | --- |
| A | Framing of the Time Table |  |  |  |
| B | Delivering the content through  the following Teaching- Learning  methods |  |  |  |
|  | a. Didactic lecture without interaction |  |  |  |
|  | b. Didactic lecture with interaction |  |  |  |
|  | c. Group seminar |  |  |  |
|  | d. Demo. only observation[ obsv] |  |  |  |
|  | e. Demo. Obsv +hands on experience |  |  |  |
|  | f. Dissection |  |  |  |
|  | g. Case Based Learning |  |  |  |
|  | h. Hospital Visit without Patient contact |  |  |  |
|  | i. Hospital Visit + Patient Contact. |  |  |  |
| C. | Assessment of students |  |  |  |
|  | a. Theory |  |  |  |
|  | b. Practical |  |  |  |
| D. | Evaluation of the entire ILP |  |  |  |

1. State your **level of satisfaction** with the following activities :

|  | Item | Satisfied to a great extent | Satisfied to some extent | Not at all satisfied |
| --- | --- | --- | --- | --- |
| 1 | Framing of timetable |  |  |  |
| 2. | Delivery of content |  |  |  |
| 3. | Assessment of Students |  |  |  |
| 4 | Evaluation of program |  |  |  |

1. State whether you **liked or disliked** the following and also **mention one point why you liked or disliked the same :[ write your comments in the respective columns below]**

|  | Item | Liked | Did not like | Uncertain |
| --- | --- | --- | --- | --- |
| 1 | Interdepartmental discussions amongst basic science faculty |  |  |  |
| 2. | Interdepartmental discussions amongst basic and clinical science faculty |  |  |  |
| 3. | Integrated Assessment of Students |  |  |  |
| 4 | Coordination and group activity towards implementation of ILP |  |  |  |

1. Having undergone this experience of implementation of ILP[CNS], **would you recommend** integration for all systems in Basic Science curriculum ? [ encircle the choice]

A] Strongly B] To some extent C] Not at all

If you feel that it can be done to some extent, then please specify the systems.

1. Give your **opinion regarding the following** as per the scale provided : [ tick the appropriate]

**1= Strongly Agree 2= Agree 3= Strongly Disagree 4= Disagree 5= Uncertain**

|  | **Item** | **1** | **2** | **3** | **4** | **5** |
| --- | --- | --- | --- | --- | --- | --- |
| **1.** | ILP has improved the understanding & application of basic science knowledge of first year students in health & disease |  |  |  |  |  |
| **2** | ILP will help the students perform better in Nervous system in University exam [Sept/Oct-07] |  |  |  |  |  |
| **3.** | ILP will help the students perform better in later days of their clinical exposure |  |  |  |  |  |
| **4.** | The concept of ILP is interesting |  |  |  |  |  |
| **5.** | ILP has helped me develop positive attitude towards medical education |  |  |  |  |  |
| **6.** | I developed better interpersonal skills in terms of listening, giving, discussing, receiving criticism |  |  |  |  |  |
| **7.** | ILP requires too much of time and is not worth all the efforts |  |  |  |  |  |
| **8.** | ILP helped me develop the ability to accept and work with faculty of different nature and subject background |  |  |  |  |  |

Kindly **give your comments** on the usefulness of integrated learning in basic sciences in the first year.
